# Supplementary figures and images for: Basic biology education in high school and acceptance of genetically modified food in Japan
Source: PLoS One. 2023 Feb 6;18(2):e0281493. doi: 10.1371/journal.pone.0281493 (PMC9901761; doi:10.1371/journal.pone.0281493)

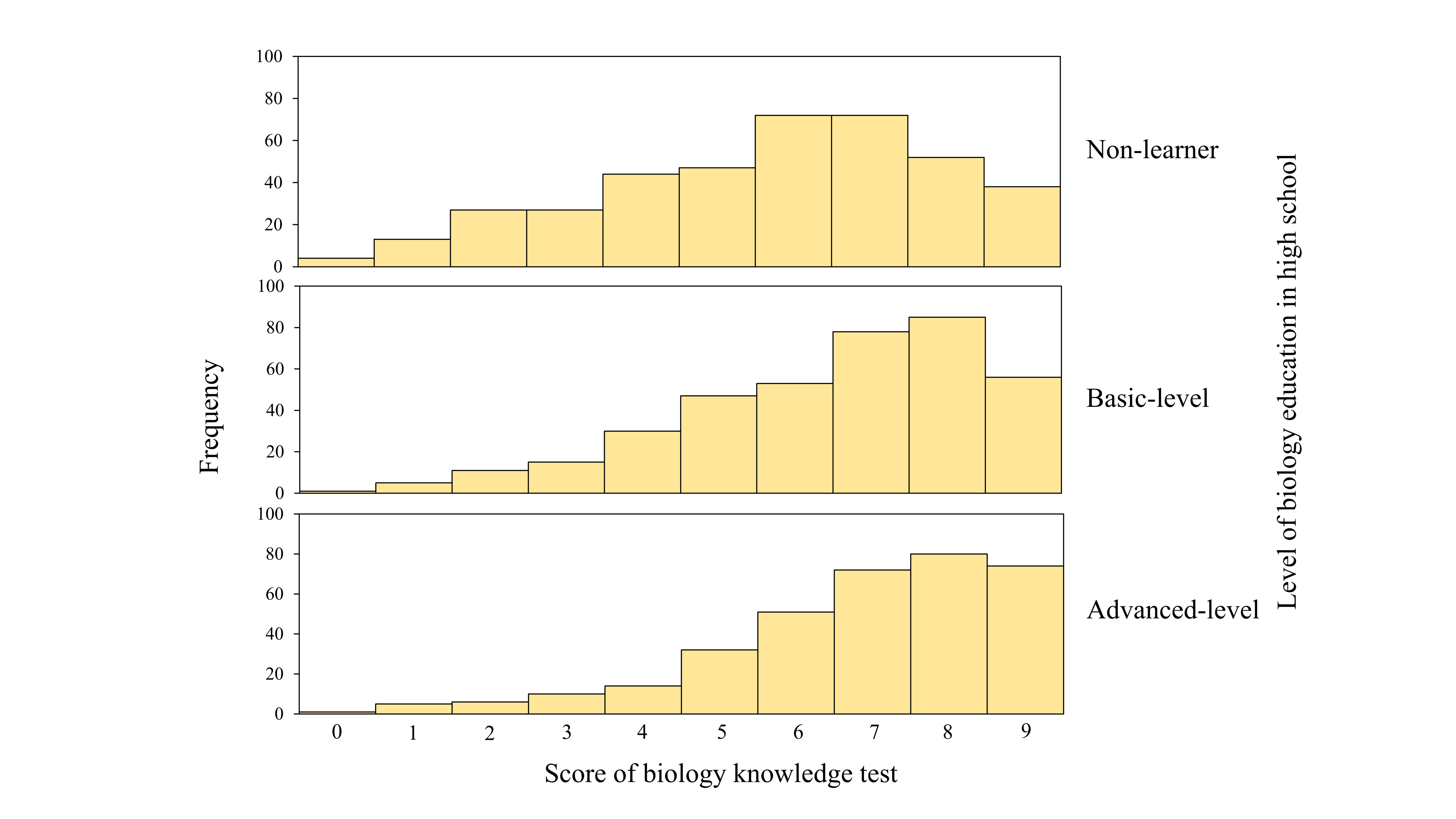

Supplement: S1 Fig — (TIF) [file pone.0281493.s001.tif]
