# Supplementary material for: Basic biology education in high school and acceptance of genetically modified food in Japan
Source: PLoS One. 2023 Feb 6;18(2):e0281493. doi: 10.1371/journal.pone.0281493 (PMC9901761; doi:10.1371/journal.pone.0281493)
Supplement: S1 Table — (DOCX) [file pone.0281493.s002.docx]

**S1 Table. Differences in Biology Textbook Content by Text Version and Level**

|  | **Textbook version** | | | | | |
| --- | --- | --- | --- | --- | --- | --- |
|  | **Version used from 2003 to 2012** | | **Version used from 1994 to 2003** | | **Version used from 1982 to 1994** | |
|  | **Level 1** | **Level 2** | **Level 1** | **Level 2** | **Level 1** | **Level 2** |
| **Content** |  |  |  |  |  |  |
| Basic information about genetics and DNA | 3 | 7 | 1 | 6 | 0 | 6 |
| Mechanism of DNA replication | 0 | 6 | 1 | 4 | 0 | 0 |
| Mechanism of gene expression | 0 | 20 | 1 | 10 | 1 | 4 |
| Gene recombination | 1 | 21 | 1 | 4 | 0 | 0 |
| Problems of biotechnology | 0 | 9 | 0 | 0 | 0 | 0 |
| **Total** | 4 | 63 | 4 | 24 | 3 | 13 |

In the textbook content analysis, we compared the numbers of instances of specific content in the textbooks published by Tokyo Shoseki Co., Ltd. We determined how many of the following concepts were included in each textbook:

Basic information about genetics and DNA: DNA; nucleotide; base; base complementarity; genome; base sequence; genetic information; and association between gene, DNA, and genome.

Mechanism of DNA replication: DNA and chromosomes, distribution of genomic information by somatic cell division, DNA replication, semiconservative replication, DNA polymerase, replication error.

Mechanism of gene expression: central dogma, functions of proteins in the body such as enzymes and antibodies, RNA, transcription, reverse transcription, operons, promoters, RNA polymerase, mutations, DNA polymorphisms, functions and types of RNA, sense and antisense strands of DNA, ribozymes, exons, introns, splicing, selective splicing, deoxyribose and ribose, translation, anticodon, triplet, codon, selective gene expression, regulatory proteins and cell differentiation, regulation of gene expression by hormones, regulatory proteins and transcription start sequences.　Gene recombination: genetic recombination, restriction enzymes, DNA ligase, vectors, transformation, cloning, PCR method, DNA sequencing, gene transfer to multicellular organisms, transgenics, GFP protein, RNA interference, genetic recombination experiments using E. coli, recombinant DNA experiments using baker's yeast, breeding, grafting, next-generation plant-breeding techniques, self-cloning, natural occurrence, methyl group, methylation, and zinc finger nuclease.

Problems of biotechnology: use of fertilized eggs for ES cells, iPS cells, human cloning, alternative organs, human genome and privacy, overuse of pesticides, and effects of introduced genes.
