# Supplementary material for: Basic biology education in high school and acceptance of genetically modified food in Japan
Source: PLoS One. 2023 Feb 6;18(2):e0281493. doi: 10.1371/journal.pone.0281493 (PMC9901761; doi:10.1371/journal.pone.0281493)
Supplement: S2 Table — (DOCX) [file pone.0281493.s003.docx]

**S2 Table. Questions on Basic Knowledge of DNA and Digestion.**

| Q1–Q3 | Knowledge of DNA | Fill in the blanks, 1 point each, total 6 points |
| --- | --- | --- |
| Q4–Q5 | Knowledge of digestion and absorption of food | Fill in the blanks, 1 point each, total 2 points |
| Q6 | Knowledge of digestion and absorption of GM food | Multiple-choice question, total 1 point |

| Read the following sentences about genetic modification and choose the word that fills in the blank from the options. | | |
| --- | --- | --- |
| Q1 | The human body is made up of about 60 trillion (a), and (a) is the smallest unit of life. | |
|  | (a) can be divided into two main parts: the nucleus and the rest of the cytoplasm. | |
|  | Inside the nucleus is (b), which is folded and shortened during cell division. | |
| Q2 | (b) is made up of a chain of (c). The chain was revealed to be a (d) structure by Watson and Crick. | |
|  | DNA is composed of four types of (e), and the order of (e) determines the properties of genes. | |
| Q3 | There are differences between human individuals, for example, ABO blood types, or frizzy and straight hair. | |
|  | These shapes and characteristics are called traits. One set of genes is referred to as (f). | |
|  | In 1990, the Human (f) Project was initiated, which revealed that humans have about 22,000 genes. | |
|  | Please choose the following that you think apply to (a). | DNA, gene, protein, cell |
|  | Please choose the following that you think apply to (b). | Chromosomes, Golgi, Endoplasmic reticulum, Ribosomes |
|  | Please choose the following that you think apply to (c). | Protein, RNA, DNA, Phosphoric acid |
|  | Please choose the following that you think apply to (d). | Single strand, double helix, triple helix, quadruple helix |
|  | Please choose the following that you think apply to (e). | Vector, Base, RNA, Genome |
|  | Please choose the following that you think apply to (f). | Exon, Operon, Codon, Genome |
| Read the following passage on digestion and absorption and choose the word that fills in the blank from the options. | | |
| Q4 | When humans eat meat, protein, which is the main component of meat, is broken down in the stomach and intestines with the help of (g). | |
| Q5 | Proteins are broken down into (h) by (g) and absorbed. |  |
|  | Please choose the following that you think apply to (g). | Digestive enzymes, ATP, chloroplasts, DNA |
|  | Please choose the following that you think apply to (h). | Glucose, glycerol, dextrin, amino acid |
| Read the following passage on digestion and absorption and choose the correct sentence. | | |
| Q6 | How do you think GM foods are digested and absorbed in your body? | |
|  | Recombinant DNA is absorbed as is and becomes protein, etc. that remains in the body and is passed on to children. | |
|  | Recombinant DNA is absorbed as is and becomes protein, etc. that remains in the body and is not passed on to children. | |
|  | Recombinant DNA is absorbed as is and becomes protein, etc. that is eliminated from the body after a long time. | |
|  | Recombinant DNA is degraded during the digestion and absorption process and becomes protein, etc. It does not remain in the body in its original form. | |
