# Supplementary material for: Basic biology education in high school and acceptance of genetically modified food in Japan
Source: PLoS One. 2023 Feb 6;18(2):e0281493. doi: 10.1371/journal.pone.0281493 (PMC9901761; doi:10.1371/journal.pone.0281493)
Supplement: Attachment — Submitted filename: Review Report.docx [file pone.0281493.s005.docx]

13^th^ November 2022,

The Editor,

Review of the manuscript entitled “Basic biology in high school and acceptance of genetically modified food in Japan; PONE-D-22-25380”

The article discusses a timely topic of whether the individual experience in biology education could influence people’s acceptance of genetically modified (GM) foods. The article has been well written, orderly and describes relevantly to the topic. Appropriate statistical procedures have been utilized, and have identified potential limitations of the study for further improvements, which can be highlighted as positive remarks of the present manuscript. Only minor concerns are highlighted herewith.

Minor comments

1. The article must be processed following the journal style for both format and the references. Pay attention to the English grammar errors. Some of the mistakes have been highlighted here.
2. Does the online questionnaire survey cover entire Japan or only the regional population of a particular urban region? Should it be mentioned in the discussion section?
3. In the materials and methods section, please support the statistical procedures with appropriate references.
4. Since the outcome of the study demonstrate that education alone may not be sufficient enough to consumer willingness to utilize GM foods, how the future education programs should improve to increase the consumer's attitude toward GM foods? Authors input can be important for both policy makers and future research studies.

English corrections

5. Line 27: Lay people; remove the gap between two words.
6. Line 62: “change food have increased”; replace have with has.
7. Line 63: “Curriculum”; change to curricula.
8. Line 85: Add “was” after GM food.
9. Line 91: Trust were embedded; change “were” to “was.
10. Line 116: Comprising of those; Remove “of”.
11. Line 120: People from all ages; change “from” to “of”.
12. Line 120: ten year ranges; change ranges to range.
13. Line 139: daily food cooked; change “cooked” to “cook”.
14. Line 204: four components were; change “were” to “was”.
15. Line 213: Differences of the Content; change “of” to “in”.
16. Line 219: Difference of Knowledge; change “of” to “in”.
17. Line 244: in the intentions; change “intentions” to intention.
18. Line 263: Additional Analysis on the; change “on” to “of”.
19. Line 267: Add “the” between “of” and “variance”.
20. Line 268: acceptance difference by; change “difference” to “differences”.
21. Line 277: change “everyday” to “every day”.
22. Line 286: schools influence the; change “influence” to “influences”.
23. Line 290: GM foods have reported; change “have” to “has”.
24. Line 315: had the lower; change “the” to “a”.
25. Line 316: GM foods than; add “more” after “GM”.
26. Line 324: may be is a one-word “maybe”.

With the above-mentioned corrections, I propose this study as a suitable one for publishing in PLOS ONE journal.

Thank you,

Amal Senevirathne (Ph.D.),

College of Veterinary Medicine,

Chungnam National University,

South Korea.
