# Supplementary material for: Basic biology education in high school and acceptance of genetically modified food in Japan
Source: PLoS One. 2023 Feb 6;18(2):e0281493. doi: 10.1371/journal.pone.0281493 (PMC9901761; doi:10.1371/journal.pone.0281493)
Supplement: Attachment — Submitted filename: Response_to_Reviewers.docx [file pone.0281493.s006.docx]

January 22, 2023

Dr. Muhammad Shahzad Aslam

Academic Editor

*PloS One*

Dear Dr. Muhammad Shahzad Aslam:

We wish to re-submit our manuscript titled “Basic biology education in high school and acceptance of genetically modified food in Japan.” The manuscript ID is PONE-D-22-25380.

We thank you and the reviewers for the time and effort you have dedicated to providing insightful feedback. The manuscript has benefited from these suggestions. We have incorporated changes into the revised manuscript that reflect the detailed suggestions you have graciously provided. We hope that our edits and the responses we have provided below satisfactorily address all the issues and concerns that you and the reviewers have noted. I look forward to working with you and the reviewers to move this manuscript closer to publication in *PloS One.*

To facilitate your review of our revisions, the following is a point-by-point response to all editor and reviewer questions and comments.

Thank you for your consideration. I look forward to hearing from you.

Sincerely,

Akihiro Mine

Nara Medical University Department of Public Health, Health Management and Policy

840 Shijo-Cho, Kashihara, Nara, 634-8521, Japan

Tel. +81-744-22-3051 Ext. 2224

Fax. +81-744-22-0037

[akihiromn777@gmail.com](mailto:akihiromn777@gmail.com)

Responses to Dr. Muhammad Shahzad Aslam

Thank you very much for your comments. Following your advice, we revised the manuscript based on the suggestions made by Reviewer 3. To follow their comment, we added reference 26 in the manuscript, and the numbers of the subsequent references have been shifted accordingly. Please see the revised manuscript.

Responses to Reviewer #1:

We thank very much for the thoughtful review.

Responses to Reviewer #2:

Thank you again for your valuable feedback. We agree with your suggestion that the independent variable regarding the biology education curriculum is identified by the age of participants, making it difficult to distinguish whether its effect on the dependent variable (acceptance of GM food) is due to differences in curriculum or differences in experiences with GM foods outside of their high school education. However, we still believed that it would be difficult to find a valid additional analysis method in the current study design. We hope to address this in our future work.

Responses to Reviewer #3:

Thank you very much for your detailed comments. Please see our point-by-point responses below.
Comment 1: Line 114 should read "Materials and Methods" and not "Material and Methods".

Response 1: Thank you for your suggestion. We have corrected this point in line 114.

Comment 2: Lines 118-124 explains more of how data collection was done and the duration of the pilot study which does not really answer my comment number 5 of the last review. What I actually meant was your readers all over the world will be interested in knowing a little more about Japan (your study area) i.e., the total population, the population composition, climate, the soil fertility status, total surface area, socio-political atmosphere etc. So maybe you create a sub-topic under "Materials and Methods" most probably the first before "Data Collection" and caption it "Description of the Study area/setting" where you briefly explain the aforementioned.

Response 2: Thank you for your careful explanation of your previous point. Following your advice, we both added more information about the duration of the pilot study in lines 138-140, and then made a sub-topic titled, “Description of the study area,” and briefly summarized information about Japan (lines 116-135).

Comment 3: About the sample size as I previously mentioned in the last review (comment 6), I'm okay with the response you gave but please do this explanation as clearly as possible under "Materials and Methods" showing the link between this work and previous works because your readers will not always have to do the kind of research you directed me to in order to understand how you came about your sample size or else it remains a myth. Moreover, you should make mention of references 2 and 3 and not just 26-31 here. Also, the adjustment from 500 to 400 questionnaires and the 1122 valid responses from 1594 should be mentioned under "Materials and Methods" and not just appearing under "Results".

Response 3: We agree with you and have added additional information about sample size and data collection in lines 146-154.

Responses to Reviewer #4:

Thank you for your thoughtful assessment and recommendation.

Responses to Reviewer #5:

Thank you very much for your acceptance.
